# Testing to Detect *Candida auris* Colonisation After Intrahospital Transfer From an Endemic Area, a Prospective Observational Study

**DOI:** 10.1111/myc.70138

**Published:** 2026-02-14

**Authors:** Laura Mezzogori, Martina Bavastro, Laura Magnasco, Federica Centorrino, Riccardo Schiavoni, Federica Portunato, Daniele Roberto Giacobbe, Antonio Vena, Vincenzo Di Pilato, Ramona Barbieri, Andrea Orsi, Giancarlo Icardi, Anna Marchese, Matteo Bassetti, Malgorzata Mikulska

**Affiliations:** ^1^ Department of Health Sciences University of Genoa Genoa Italy; ^2^ Infectious Diseases Unit, IRCCS Ospedale Policlinico San Martino Genoa Italy; ^3^ Department of Surgical Sciences and Integrated Diagnostics (DISC) University of Genoa Genoa Italy; ^4^ IRCCS Ospedale Policlinico San Martino, Microbiology Unit Genoa Italy; ^5^ IRCCS Ospedale Policlinico San Martino, Hygiene Unit Genoa Italy

**Keywords:** *Candida auris*, candidemia, colonisation, intensive care unit, screening, surveillance, swabs, transmission prevention

## Abstract

**Background:**

Current international guidelines lack clear recommendations on the management of non‐colonised patients undergoing intra‐hospital transfer from the ward in which horizontal transmission of *Candida auris* is known to occur (defined as endemic for *Candida auris*) to wards with no horizontal transmission detected (defined as non‐endemic wards), particularly regarding the timing and number of screening swabs needed to exclude colonisation.

**Methods:**

Single‐center prospective observational study at a tertiary‐care hospital in Genoa, Italy, including adults transferred from the *C. auris* endemic‐ICU (eICU) to non‐endemic wards between January and December 2024. Patients who tested negative for *C. auris* colonisation both at eICU admission and at transfer, and who had ≥ 1 screening swab performed post‐transfer, were included. Swabs (bilateral axilla/groin) were performed on Days 0–1, 2–3 after transfer, and then weekly, and tested for *C. auris* with real‐time PCR. Patients were considered sufficiently screened to exclude colonisation if they underwent ≥ 2 swabs within the first 4 weeks after transfer.

**Results:**

Among 462 patients transferred from the eICU, 440 (95.2%) were non‐colonised. Among them, 275 (62.5%) met inclusion criteria, and 208 (75.6%) were considered sufficiently screened. *C. auris* colonisation was detected in 34/208 (16.3%) patients, with 21 (61.8%) positive in the first post‐transfer swab. Among 99 patients who had a negative result of a swab performed within 1 day before transfer, 7 (7.1%) resulted later positive. *C. auris* candidemia occurred in 4/34 (11.8%) patients with colonisation detected post‐transfer, compared to 1/35 (2.9%) patients found colonised during eICU stay, and none occurred in non‐colonised individuals.

**Conclusions:**

A single negative screening test at eICU discharge is insufficient to exclude colonisation, even if performed within 24 h from transfer. Repeated screening, ideally within the first 2 weeks post‐transfer, is essential to detect colonisation and prevent further *C. auris* transmission.

## 1 Introduction

The World Health Organization [1] and the Centers for Disease Control and Prevention [2] have included *Candida auris* among the highest‐priority fungal pathogens of public health concern.

The ability of *C. auris* to persist on the skin for prolonged periods [3], combined with its capacity to spread through contact with contaminated surfaces or direct skin‐to‐skin transmission, facilitates its dissemination within healthcare settings [4]. An estimated 5%–25% of colonised individuals progress to invasive infections, which are often associated with high mortality rates [5, 6, 7].

*C. auris* cases have to date been documented in over 50 countries across six continents [4, 8]. In most countries [9, 10, 11, 12, 13, 14, 15, 16], its dissemination peaked during the COVID‐19 pandemic in 2020. Prolonged hospital stays, underlying comorbidities, use of broad‐spectrum antibiotics, immunosuppressive therapy, mechanical ventilation, and the presence of indwelling urinary and intravenous catheters facilitated the persistence of *C. auris* specifically in intensive care units (ICU) [17, 18, 19]. Moreover, beyond patient‐related risk factors, the pandemic may have influenced infection prevention and control measures facilitating the spread of the pathogen [19, 20]. Notably, repeated outbreaks of *C. auris* in long‐term care facilities contribute to its persistence and transmission [21].

In our hospital, an outbreak of *C. auris* was recorded during the COVID‐19 pandemic [22], with more than 90% of all new *C. auris* cases occurring in two of five ICUs, where horizontal transmission of *C. auris* continued despite strict infection control measures, even though the rate of patients acquiring colonisation during ICU stay decreased overtime. We defined these ICUs as endemic for *C. auris* (eICU), *and one of them was closed after* the pandemic's resolution [6]. A similar confined distribution of cases of *C. auris* transmission has been observed in Greece, with a long‐lasting outbreak occurring in selected ICUs within a tertiary care hospital [23].

Although some recommendations exist for *C. auris* outbreak prevention, current guidelines often lack specific indications regarding screening practices. Most protocols focus on patients admitted from known endemic facilities or with known risk factors for colonisation, but do not clearly address the management of patients transferred within the same hospital from an endemic unit to a non‐endemic one, particularly when a prior negative screening is performed immediately before transfer.

In response to the outbreak in our hospital, an internal screening protocol was introduced in 2020 and subsequently refined to enhance post‐transfer testing from eICU.

While cutaneous swabbing is widely recommended, there is no consensus on the optimal number, anatomical site, or timing for testing. Practices vary significantly: some protocols suggest three swabs taken 24 h apart, others recommend screening with one swab upon admission followed by weekly tests, but in many cases, testing intervals remain undefined [24, 25, 26, 27, 28]. Similarly, the optimal duration of preventive contact precautions after negative screening results remains unclear. Understanding the optimal screening protocols and isolation practices for patients at risk is crucial to minimize undetected colonisation and limit further transmission of *C. auris* within healthcare facilities.

Our study aimed to investigate the optimal duration and frequency of screening for *C. auris* colonisation in patients transferred as non‐colonised from eICU.

## 2 Materials and Methods

### 2.1 Study Design and Objectives

This was a single‐center, prospective study conducted at a tertiary‐care medical center in Genoa, Italy, from January 1st, 2024, to December 31st, 2024. Follow up of patients transferred in the late months of 2024 was extended up to the end of March 2025.

The primary objective of this study was to investigate the optimal frequency and duration of screening for *C. auris* colonisation in patients transferred as non‐colonised from the only eICU. Indeed, of two eICUs in our institution, one caring specifically for COVID‐19 patients had been closed after the end of the pandemic and was no longer in use during the study period. The secondary objective was to evaluate the prevalence of *C. auris* candidemia in colonised patients during the study period.

For the primary objective, inclusion criteria were: age ≥ 18 years, transferred alive from eICU, at least one negative screening performed in the eICU, and at least one screening performed in a non‐endemic ward.

All patients included in this analysis had tested negative for *C. auris* colonisation at all screenings performed during their eICU admission, allowing the evaluation of new colonisation events occurring after transfer to non‐endemic wards.

For the secondary objective, we identified all colonised patients during the study period who developed *C. auris* candidemia.

### 2.2 Definitions and Screening Protocols

Colonisation with *Candida auris* was defined as its detection in cutaneous swabs performed in axillae and groin bilaterally (with molecular and/or conventional culture techniques), as well as in urine and/or respiratory samples (tracheal aspirates or bronchoalveolar lavage samples for patients undergoing mechanical ventilation). Whenever *C. auris* was detected from non‐cutaneous sites, molecular cutaneous swabs were also performed to assess skin colonisation.

Screening for cutaneous colonisation was performed using a commercially available real‐time PCR assay (Progenie Molecular, Spain).

*C. auris* candidemia was defined as the isolation of *C. auris* from blood cultures (MALDI‐TOF MS—VITEK MS; bioMe'rieux, Marcy‐l'Etoile, France), identified using VITEK MS v4.0 software.

According to local screening protocol, introduced in 2020, patients admitted to all ICUs in our hospital underwent weekly screening to monitor *C. auris* colonisation status. eICU was the only ICU (among the 4 ICUs serving our hospital during the study period) with ongoing *C. auris* transmission, characterised by a sustained detection of new cases of colonisation over time. No other wards outside that eICU were considered endemic in our institution. Importantly, this eICU continued to admit all individuals according to clinical needs, but given a possibility of horizontal transmission, all admitted patients were repeatedly screened (at admission, weekly and at discharge) according to the institutional infection control protocol.

Patients were transferred from the eICU to non‐endemic wards when clinically stabile, regardless of their *C. auris* colonisation status. Upon transfer, those who had tested negative for *C. auris* colonisation during eICU stay underwent additional screening before eICU discharge and continued screening after admission to other hospital wards, with swabs collected at days 0–1, 2–3, and subsequently on a weekly basis for up to 4 weeks or until hospital discharge. This approach allowed the detection of new colonisation events occurring after transfer to non‐endemic wards (primary endpoint of the study). Contact precautions were also applied until at least 2 negative screening results were obtained.

Screening performed in non‐endemic wards was considered “sufficient” if at least two swabs were collected within the first 4 weeks after eICU transfer or if colonisation was detected at the first control swab.

### 2.3 Data Collected for the Analysis

Clinical and microbiological data were retrieved from electronic medical records. Transfers from eICU to non‐endemic wards were tracked using locally implemented business intelligence software for data integration and analysis. Screening data were reviewed at least twice weekly throughout the study period.

The Charlson Comorbidity Index [29] was recorded to assess baseline comorbidity.

Non‐endemic wards were contacted by phone by study personnel to improve adherence to the screening schedule.

### 2.4 Statistical Analysis

Categorical variables were summarised with numbers and percentages, and continuous variables with median and interquartile range. Two‐tailed *p* values were considered significant if ≤ 0.05. All analyses were performed with SPSS v.21.0 (IBM, Armonk, NY, USA).

### 2.5 Ethic

The study was conducted in accordance with the principles of the Declaration of Helsinki.

The authors confirm that the ethical policies of the journal, as outlined in the author guidelines, have been followed. The study was approved by the Ethics Committee of the Liguria Region (Protocol No. 575/2023). Informed consent for the use of clinical data for research purposes was obtained from all patients. The requirement for study‐specific informed consent was waived, as all procedures were part of the standard institutional screening protocol.

## 3 Results

### 3.1 Included Patients

**FIGURE 1:**
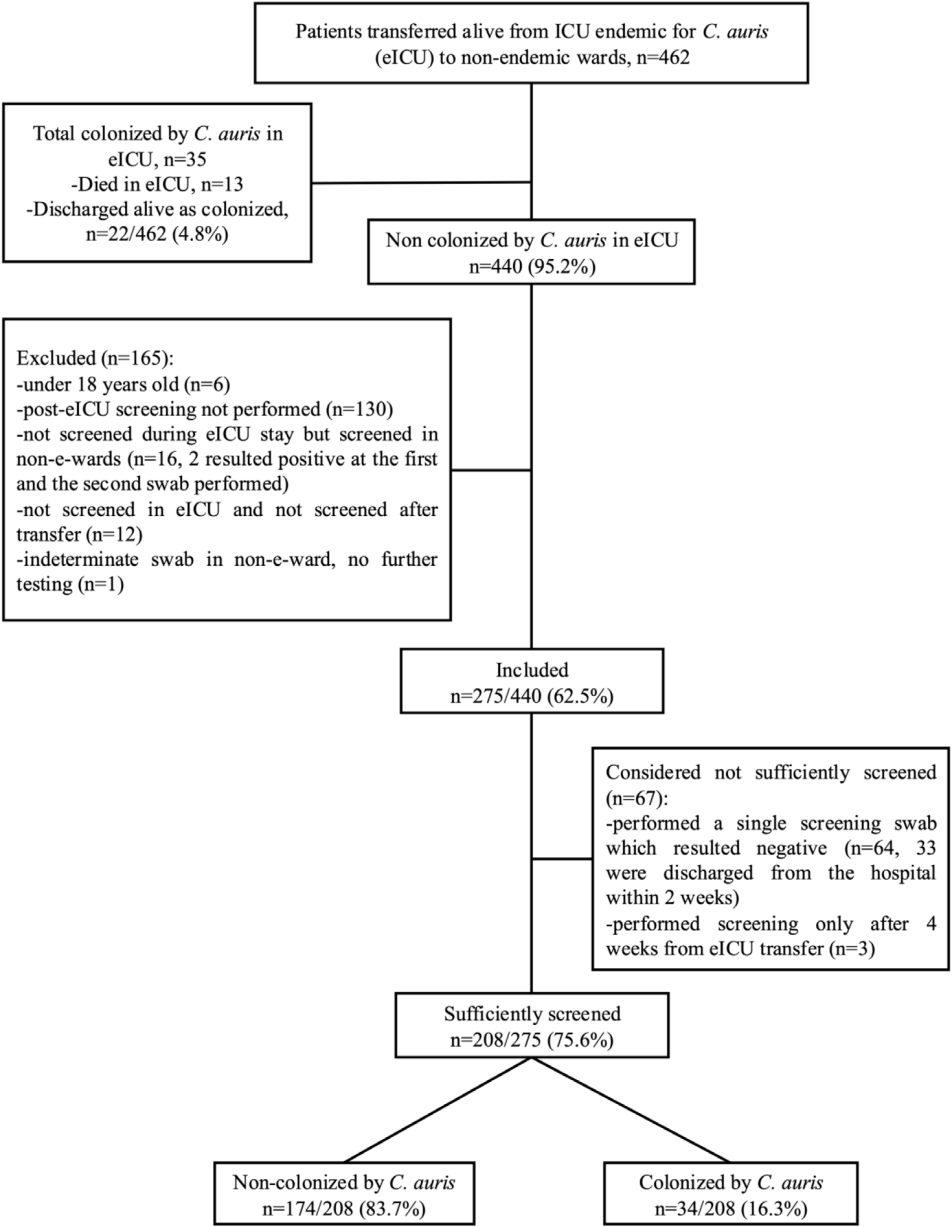
Flow chart of the study. Included: Patients who tested negative for *C. auris* colonisation both at eICU admission and discharge, and who had ≥ 1 screening swab performed post‐transfer. Sufficiently screened: Patients who underwent ≥ 2 swabs within the first 4 weeks after transfer.

During the study period 462 patients were transferred from the eICU to other wards. Of these, 440/462 (95.2%) patients were transferred as non‐colonised with *C. auris* , while 22/462 (4.8%) had tested positive during their eICU stay. Additional 13 *C. auris‐*colonised patients died during their eICU stay; for a total of 35 patients found colonised with *C. auris* while admitted to eICU during the study period. Overall, 275/440 (62.5%) patients were included in the study (see Figure 1 for reasons for exclusion). Among the 208 sufficiently screened patients (75.6% of included patients), 174/208 (83.7%) tested negative at all screenings performed, whereas 34/208 (16.3%) were found to be colonised with *C. auris* outside the eICU.

The median duration of stay in the eICU was 5 days (IQR 2.3–11.8) for the overall population, with similar values observed across subgroups. The demographic and clinical characteristics of the study population are summarised in Table 1.

**TABLE 1:** Comparison of the main demographic and clinical characteristics of the study population.

	Overall, *N* = 275	Sufficiently screened, *N* = 208	Negative, *N* = 174 (of sufficiently screened)	Positive, *N* = 34 (of sufficiently screened)	*p* for negative vs. positive
*Demographics characteristics*					
Male sex, *n* (%)	155 (56.4)	127 (61.1)	103 (59.2)	24 (70.6)	0.213
Age (years), median (IQR)	65 (54.5–75)	65 (54.8–74.5)	64.5 (55.3–74)	65.6 (50.5–73)	0.893
*Length of stay (days), median (IQR)*					
eICU length of stay	5 (2.3–11.8)	5.5 (2.5–19.1)	5 (2.2–12.8)	6 (3.5–14)	0.309
post eICU length of stay	20 (12.5–39)	23 (13.4–43.2)	22 (13.1–39)	32 (14.7–52)	0.116
*Reason for admission to eICU, n (%)*					0.671
Medical	112 (40.7)	84 (40.4)	69 (39.7)	15 (44.1)	
Abdominal surgery	38 (13.8)	28 (13.5)	25 (14.3)	3 (8.8)	
Other surgery	125 (45.5)	96 (46.1)	80 (46.0)	16 (47.1)	
*Ward of discharge after eICU stay, n (%)*					0.414
Neurosurgery	46 (16.7)	43 (20.7)	33 (19.0)	10 (29.4)	
Other surgery	88 (32.0)	64 (30.8)	55 (31.6)	9 (26.5)	
Medicine	121 (44.0)	85 (40.8)	71 (40.8)	14 (41.2)	
Rehabilitation	12 (4.4)	11 (5.3)	11 (6.3)	0 (0.0)	
Other non‐endemic ICU	8 (2.9)	5 (2.4)	4 (2.3)	1 (2.9)	
Number of negative swabs performed in the eICU, median (IQR)	1 (1–2)	1 (1–2.5)	1 (1–3)	1 (1–3)	0.825
Last swab performed within 3 days from discharge, *n* (%)	180 (65.4)	132 (63.5)	113 (64.9)	19 (55.9)	
Last swab performed within 2 days from discharge, *n* (%)	145 (52.7)	106 (51.0)	93 (53.4)	13 (38.2)	
Last swab performed within 1 day from discharge, *n* (%)	99 (36.0)	73 (35.1)	66 (37.9)	7 (20.6)	
*Follow up*					
Discharged from hospital alive, *n* (%)	264 (96.0)	199 (95.7)	166 (95.4)	33 (97.1)	0.664

Abbreviations: eICU, Endemic ICU; IQR, Interquartile range.

**FIGURE 2:**
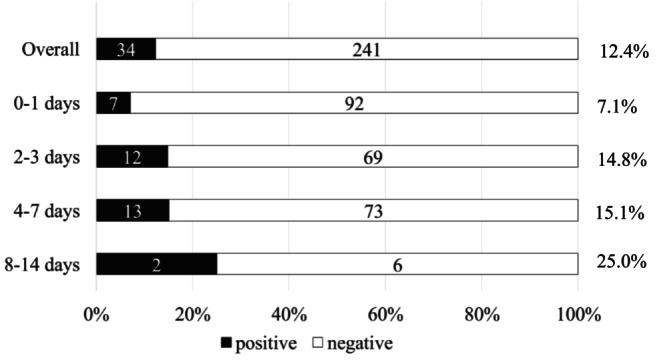
Rate of detection of *C. auris* colonisation after transfer from eICU; overall (among included patients) and stratified according to the number of days elapsing between the last negative screening performed in the eICU and the day of transfer. Denominators refer to all patients tested at each timepoint. One patient was screened on day 16 and was not included in this figure. eICU, endemic intensive care unit.

Figure 2 represents the rate of detection of colonisation outside the eICU, stratified according to the timing of last screening performed while admitted to eICU. Even in case of a negative swab performed within 24 h before transfer, post‐transfer colonisation was detected in 7.1% of tested patients (7/99).

### 3.2 Overall Detection of *C. auris* Colonisation

**FIGURE 3:**
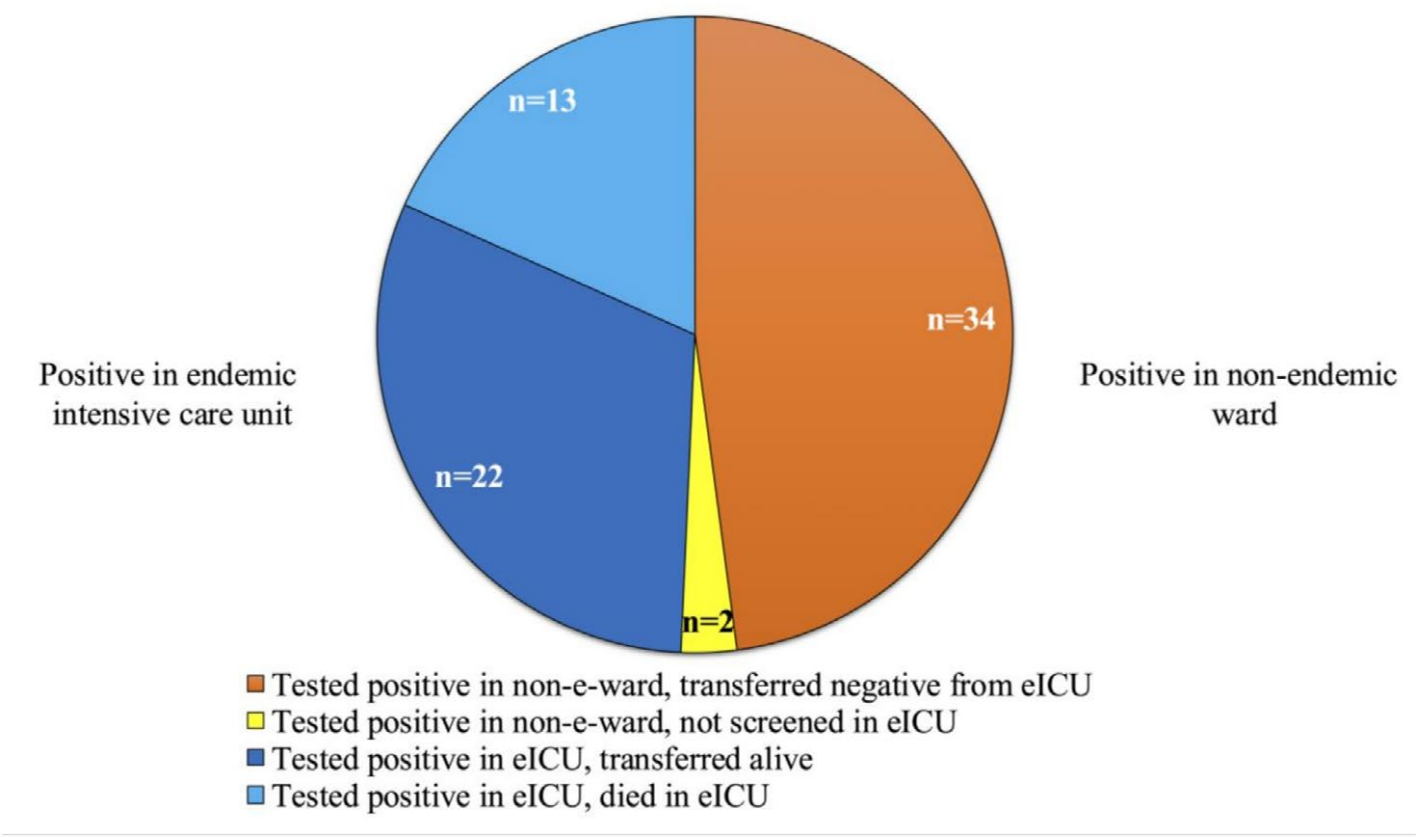
Acquisition of *Candida auris* colonisation among the 71 positive patients during the study period. eICU, endemic ICU; non‐e‐ward, non‐endemic ward.

During the study period, a total of 71 patients were identified as colonised with *C. auris* . Of these, 35 (49.3%) patients acquired colonisation in the eICU, while 34 (47.9%) were identified after transfer from the eICU, and additional 2 (2.8%) were identified as colonised after transfer from eICU, but did not perform any screening while in eICU (Figure 3).

All patients were colonised at skin level, while 13/69 (18.8%) also showed colonisation at additional sites, including the urinary tract (8/69, 11.5%) and the respiratory tract (6/69, 8.7%). Table 2 summarises the main characteristics of patients colonised with *C. auris* during the study period.

**TABLE 2:** Comparison of the main demographic and clinical characteristics of the patients colonised by *Candida auris* during the study period.

Variable	Overall, *N* = 69	Positive in eICU, *N* = 35	Positive in non‐e‐ward, *N* = 34	*p*
*Demographics characteristics*				
Male sex, *n* (%)	51 (73.9)	27 (68.6)	24 (70.6)	0.535
Age (years), median (IQR)	65 (56–73)	63 (56–72)	65.6 (50.5–73)	0.778
eICU lengh of stay (days), median (IQR)	14 (5.9–26)	21 (14–51.5)	6 (3.5–14)	< 0.001
Time to colonisation following admission to the eICU (days), median (IQR)	—	9 (6–20)	—	—
Time to colonisation following eICU discharge (days), median (IQR)	—	—	1.5 (1–6)	—
Other colonisation sites, *n* (%)	13 (18.8)	9 (25.7)	4 (11.8)^a^	
Urinary tract	8 (11.5)	5 (14.3)	3 (8.8)	0.506
Respiratory tract	6 (8.7)	4 (11.4)	2 (5.9)	0.853
*Follow up*				
Discharged from hospital alive, *n* (%)	52 (75.4)	19 (54.3)^b^	33 (97.1)	0.002

Abbreviations: eICU, endemic intensive care unit; IQR, interquartile range; non‐e‐ward, non‐endemic ward.

^a^ 1 patient had both localization; number repeated in each group.

^b^ 13/16 died in eICU.

### 3.3 Time to Detection of Colonisation

Of the 34 patients who became colonised after transfer from the eICU, 21 (61.8%) tested positive on their first swab (16 within 1 day, 4 between 3–7 days, and 1 after 10 days); 8 (23.5%) on their second swab (7 between 1–3 days, and 1 after 10 days); and 4 (12.9%) on their third swab (on Days 8, 20, 22, and 38). Among the 79 patients who had been screened by Day 8 with 3 swabs, one (1.3%) tested positive on Day 8. Overall, among 30 patients who had been screened with 5 swabs, one tested positive (3.3%) for the first time on the fifth swab performed 40 days after eICU transfer.

Among 17 patients who were excluded due to the absence of an eICU screening swab but were screened after transfer, two resulted positive: one on the first swab performed within 48 h after eICU transfer and the other on Day 7 after an initial negative swab collected on the day of transfer (detected colonisation rate: 2/17, 11.8%).

**FIGURE 4:**
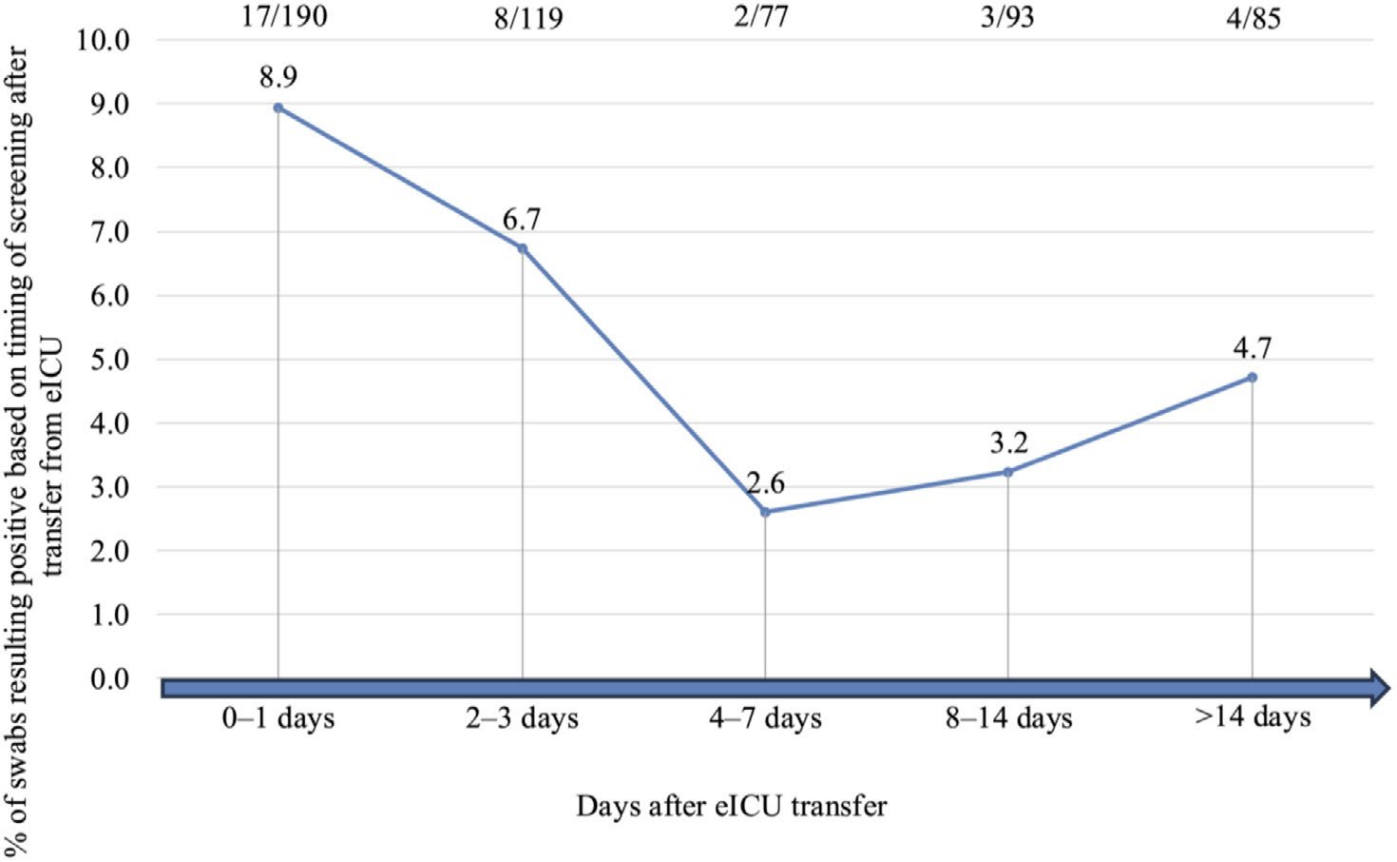
Detection of *C. auris* colonisation rate after endemic intensive care unit (eICU) transfer to non‐endemic ward among included patients and stratified on the basis of days elapsed between transfer and the day of the positive result in a non‐endemic ward. Not all patients underwent screening at all pre‐defined time points.

The detection rate of *C. auris* colonisation overtime after transfer from the eICU is reported in Figure 4.

### 3.4 *C. auris* Candidemia

During the study period, among the 34 patients who became colonised after transfer from the eICU, 4 (11.8%) developed candidemia. The time from colonisation detection to candidemia onset was 2, 24, 49, and 50 days.

Conversely, among the 35 patients who were already colonised during their stay in the eICU, with a median follow‐up of 47 days (IQR 19.5–113.5), only one case of candidemia was observed (2.9%), which occurred 16 days after colonisation detection, when the patient was still admitted to eICU.

No cases of *C. auris* candidemia occurred in patients who were not colonised with *C. auris* , with a total number of hospital admissions during the study period of 47,861.

Overall, the proportion of *C. auris* candidemia among colonised patients during the study period was 7.3%, with lower rate in patients colonised during the eICU stay compared to those with colonisation detected after transfer (2.9% versus 11.8%, *p* = 0.15).

Interestingly, we observed a notable difference in the prevalence of candidemia between the clinical characteristics of patients with *C. auris* candidemia are summarised in Table 3.

**TABLE 3:** Characteristics of patients with *Candida auris* candidemia.

Variable	Candidemia in patients colonised in non‐e‐ward				Candidemia in patient colonised in eICU
Patient	1	2	3	4	5
Sex	Male	Male	Female	Male	Male
Age, years	75	74	88	65	41
Charlson comorbidity index	3	6	6	4	1
Reason for admission to eICU	Polytrauma with spinal cord injury	Cranial trauma with acute subdural hematoma	Cranial trauma with acute subdural hematoma	Cholecystitis after chemotherapy	Recurrent pulmonary embolism
eICU length of stay, days	8	57	8	5	73
Time from eICU discharge to detection of colonisation, days	1	3	1	3	Not applicable Days from eICU admission to colonisation: 11
Time from colonisation to candidemia, days	2	29	49	50	15
Other colonisation sites	None	None	None	Respiratory and urinary tract	Respiratory tract
Time from candidemia to death, days	—	155	124	42	—
Comments	Repeated ICU and non‐ICU ward transfer; multiple infectious complications and their treatment; second episode of *C. auris* candidemia after 28 days from the first episode	Repeated hospitalizations due to cardiac, neurological and infectious complications. Death due to *Achromobacter xylosoxidans* BSI	Repeated antibiotic therapies; recurring seizures; transferred alive to long term care facility.	Repeated hospital admissions for treatment of cerebral lymphoma; death due to lymphoma progression	Discharged alive from ICU 47 days after candidemia onset

Abbreviations: BSI, blood stream infection; eICU, endemic Intensive Care Unit; non‐e ward, non‐endemic ward.

## 4 Discussion

Our study is the first one to provide organised data on the screening for *Candida auris* colonisation in patients transferred from an endemic to a non‐endemic ward within the same hospital.

Current international recommendations suggest screening for *C. auris* colonisation in individuals at high risk, particularly patients recently admitted to healthcare facilities with known or suspected transmission of *C. auris* , or who have epidemiological links to other cases [9, 24, 25, 28]. Although defining who should be screened, guidelines provide limited and heterogenous guidance on key operational aspects, namely, the number of swabs to be performed, the type of microbiological test to perform (molecular vs. culture‐based), the need for repeated testing following a negative result, and the optimal frequency of screening. Recent studies have emphasised key aspects to consider in *C. auris* screening and infection control, including the importance of active surveillance, environmental hygiene, and targeted screening strategies in high‐risk or endemic settings [30, 31]. However, these studies mainly addressed institutional policies and general principles of outbreak containment, without providing standardised recommendations on the timing or repetition of screening.

Our study adds to this evidence by proposing a structured screening schedule and by addressing a specific epidemiological scenario, where within a single institution endemic ICU and non‐endemic wards coexist. Moreover, such intra‐hospital model effectively mirrors inter‐facility transfer dynamics, offering practical insight into colonisation detection and prevention strategies applicable at both hospital‐level and regional containment efforts.

Culture‐based screening remains the reference standard in many settings, but molecular assays offer a faster turnaround time and may enhance sensitivity, particularly in individuals with a low fungal burden or intermittent shedding of *C. auris* [32]. At our institution, PCR‐based screening was adopted as the standard method due to the aforementioned advantages, as well as to shorten the time length of contact precautions for patients testing negative.

This study provides evidence that screening only during admission and at discharge from endemic units, substantially underestimates the true burden of *C. auris* colonisation. Indeed, among patients who tested negative for *C. auris* colonisation at eICU discharge despite repeated weekly re‐testing, an additional 50% of all colonised cases were identified only after transfer. These findings suggest that screening limited to the endemic unit is insufficient and colonisation may become detectable only after eICU discharge, possibly due to acquisition during the final days or even hours of eICU stay or delayed detectability by diagnostic methods due to a progressive increase in skin burden of *C. auris*. Additionally, we observed that a single screening swab performed outside the eICU was able to detect only 60% of colonised individuals. Performing a second swab within 14 days allowed us to identify almost 90% of colonised patients. Therefore, our data support the implementation of at least twice molecular screening protocol for all patients transferred as non‐colonised from an endemic ward.

A notable aspect highlighted by our findings is the need for pre‐emptive contact isolation for patients transferred from endemic wards or facilities. Similarly, during a *C. auris* outbreak in the UK, individuals who had contact with a confirmed case were placed in pre‐emptive isolation and only released after three consecutive negative culture‐based screening swabs. Screening was then continued weekly until discharge [5]. However, no data was available on the performance, and thus clinical utility, of the second and third screening swab. Nevertheless, based on that study, national guidelines from the UK and Australia/New Zealand recommended three negative screenings, performed at least 24 h apart, before discontinuing contact isolation in at‐risk patients [24, 25]. Our data demonstrated that when endemic wards exist within the same institution, patients relocated from these areas to non‐endemic wards should be managed with infection prevention and control precautions similar to those applied during inter‐facility transfers, at least until two consecutive negative molecular swabs were obtained.

These findings carry important clinical and public health implications. Incomplete screening and premature discontinuation of isolation measures may lead to silent dissemination of *C. auris* across wards or institutions, undermining infection control efforts.

All cases of *C. auris* candidemia occurred in patients previously identified as colonised, consistent with existing literature indicating that colonisation is a prerequisite for invasive disease and further supporting the absence of cases of undetected colonisation [33]. The overall prevalence of *C. auris* candidemia among colonised patients in our hospital showed a decreasing trend compared to previous years (15%–26% during the period 2020 to 2023) [6, 20]. Interestingly, we observed higher rate of candidemia among patients with colonisation detected after transfer compared to those with colonisation diagnosed during eICU stay (11%–8% versus 2.9%). A possible explanation for such a difference might be a different survival rate among patients acquiring *C. auris* colonisation early during their ICU stay and possibly dying in ICU due to other complications, prior to candidemia development. However, this finding highlights the importance of post‐transfer screening also to provide prompt effective therapy in case of suspected infection in colonised patients, since no case of *C. auris* candidemia occurred in our hospital among non‐colonised patients.

Our study has several strengths. To our knowledge, it represents one of the most comprehensive and standardised evaluations of *C. auris* colonisation management in patients transferred from an endemic to a non‐endemic ward in the same institution. The implementation of serial screening enabled us to identify operational gaps not fully addressed by current protocols and to propose targeted interventions. This hospital organization mirrors the dynamics of patient transfers between endemic and non‐endemic institutions, making our findings relevant to similar real‐world settings.

Additionally, patient identification and data extraction were performed using software‐based selection followed by manual validation to maximise enrolment, although this approach may have missed a small number of eligible cases. The use of molecular screening likely contributed to the timely and sensitive detection of colonisation.

However, we also observed that compliance with the screening protocol was suboptimal, particularly in high‐burden settings, which may have led to underestimation of true colonisation rates. Another limitation of our study is the fact that the generalizability of our findings depends on local epidemiology, type of endemic ward and type of screening method used and may not be directly applicable to other settings without appropriate contextualization. Moreover, cutaneous screening was limited to the axilla and groin, in accordance with current recommendations, while additional anatomical sites (e.g., nasal swabs) were not actively screened. Recent evidence suggest that including these sites could improve detection sensitivity [34, 35].

To conclude, our data suggest that a more tailored approach to screening and isolation, based on local epidemiology and repeated testing, may help to optimise resource utilisation without compromising patient safety. The epidemiological duality at our institution underscores the need for internal risk stratification and unit‐specific protocols and could be a model for other large healthcare facilities facing similar challenges.

Our findings support the need for repeated screening after transfer from endemic to non‐endemic units, ideally within the first 2 weeks post‐transfer, and for maintaining pre‐emptive isolation even in patients transferred as non‐colonised, in order to effectively identify delayed colonisation and prevent further transimission.

## Author Contributions

**Laura Mezzogori:** conceptualization, investigation, validation, visualization, writing – original draft, writing – review and editing. **Martina Bavastro:** investigation, data curation, writing – review and editing. **Laura Magnasco:** conceptualization, investigation, formal analysis, visualization, validation, writing – review and editing. **Federica Centorrino:** investigation, data curation, writing – review and editing. **Riccardo Schiavoni:** investigation, data curation, writing – review and editing. **Federica Portunato:** data curation, investigation, writing – review and editing. **Daniele Roberto Giacobbe:** data curation, investigation, writing – review and editing. **Antonio Vena:** data curation, investigation, writing – review and editing. **Vincenzo Di Pilato:** data curation, investigation, writing – review and editing. **Ramona Barbieri:** investigation, data curation, writing – review and editing. **Andrea Orsi:** investigation, writing – review and editing, software. **Giancarlo Icardi:** software, investigation, writing – review and editing. **Anna Marchese:** investigation, resources, writing – review and editing. **Matteo Bassetti:** data curation, investigation, writing – review and editing. **Malgorzata Mikulska:** conceptualization, investigation, methodology, formal analysis, software, resources, visualization, supervision, writing – review and editing, writing – original draft.

## Funding

This is a non‐profit project supported by an unconditional grant awarded within the framework of the Gilead Fellowship Program 2023 [grant number N375A]. The company had no role in the design of the study, data collection or analysis, interpretation of results, or in the writing or approval of the manuscript.

## Conflicts of Interest

Outside the submitted work, Matteo Bassetti has received funding for scientific advisory boards, travel, and speaker honoraria from Cidara, Gilead, Menarini, MSD, Mundipharma, Pfizer, and Shionogi. Outside the submitted work, Daniele Roberto Giacobbe reports investigator‐initiated grants from Pfizer, Shionogi, BioMérieux, Menarini, Tillotts Pharma, and Gilead Italia, travel support from Pfizer, and speaker/advisor fees from Pfizer, Menarini, BioMérieux, Advanz Pharma, and Tillotts Pharma. Outside the submitted work, Antonio Vena reports personal fees for speaker/advisor from Pfizer Inc., Shionogi, Tillotts Pharma, Menarini, Gilead Italia, Mundipharma, Advanz pharma and MSD. Outside the submitted work, Vincenzo Di Pilato reports travel support and speaker honoraria from Arrow Diagnostics. Outside the submitted work, Anna Marchese has received research grants for the laboratory from DiaSorin. Outside the submitted work, Laura Mezzogori received an honorarium from HealthData Consulting S.r.l. for acting as a Discussant at an educational event The remaining authors declared no potential conflicts of interest with respect to the research, authorship, and/or publication of this article.

## Data Availability

The data that support the findings of this study are available from the corresponding author upon reasonable request. The provided material includes sensitive data that should only be accessed for appropriate and justified reasons, in compliance with confidentiality and ethical standards.
